# Deep transfer learning for automated single-lead EEG sleep staging with channel and population mismatches

**DOI:** 10.3389/fphys.2023.1287342

**Published:** 2024-01-05

**Authors:** Jaap F. Van Der Aar, Daan A. Van Den Ende, Pedro Fonseca, Fokke B. Van Meulen, Sebastiaan Overeem, Merel M. Van Gilst, Elisabetta Peri

**Affiliations:** ^1^ Department of Electrical Engineering, Eindhoven University of Technology, Eindhoven, Netherlands; ^2^ Philips Research, Eindhoven, Netherlands; ^3^ Kempenhaeghe Center for Sleep Medicine, Heeze, Netherlands

**Keywords:** polysomnography, sleep staging, single-channel, wearable EEG, fine-tuning, deep learning

## Abstract

**Introduction:** Automated sleep staging using deep learning models typically requires training on hundreds of sleep recordings, and pre-training on public databases is therefore common practice. However, suboptimal sleep stage performance may occur from mismatches between source and target datasets, such as differences in population characteristics (e.g., an unrepresented sleep disorder) or sensors (e.g., alternative channel locations for wearable EEG).

**Methods:** We investigated three strategies for training an automated single-channel EEG sleep stager: pre-training (i.e., training on the original source dataset), training-from-scratch (i.e., training on the new target dataset), and fine-tuning (i.e., training on the original source dataset, fine-tuning on the new target dataset). As source dataset, we used the F3-M2 channel of healthy subjects (N = 94). Performance of the different training strategies was evaluated using Cohen’s Kappa (*κ*) in eight smaller target datasets consisting of healthy subjects (N = 60), patients with obstructive sleep apnea (OSA, N = 60), insomnia (N = 60), and REM sleep behavioral disorder (RBD, N = 22), combined with two EEG channels, F3-M2 and F3-F4.

**Results:** No differences in performance between the training strategies was observed in the age-matched F3-M2 datasets, with an average performance across strategies of κ = .83 in healthy, κ = .77 in insomnia, and κ = .74 in OSA subjects. However, in the RBD set, where data availability was limited, fine-tuning was the preferred method (*κ* = .67), with an average increase in κ of .15 to pre-training and training-from-scratch. In the presence of channel mismatches, targeted training is required, either through training-from-scratch or fine-tuning, increasing performance with κ = .17 on average.

**Discussion:** We found that, when channel and/or population mismatches cause suboptimal sleep staging performance, a fine-tuning approach can yield similar to superior performance compared to building a model from scratch, while requiring a smaller sample size. In contrast to insomnia and OSA, RBD data contains characteristics, either inherent to the pathology or age-related, which apparently demand targeted training.

## 1 Introduction

Sleep stage scoring is an essential component of both sleep research and the clinical diagnosis of sleep disorders. Conventionally, polysomnographic (PSG) recordings are manually scored by trained clinicians according to the American Academy of Sleep Medicine (AASM) guidelines (Troester et al., 2023). 30-s epochs are classified as either Wake, N1, N2, N3, or rapid-eye-movement (REM) sleep through visual inspection of multiple leads, including electroencephalography (EEG), electromyography (EMG), and electrooculography (EOG). This manual process is labor-intensive, time-consuming, and suffers from a relatively large degree of inter-rater disagreement (Danker-Hopfe et al., 2004; Danker-Hopfe et al., 2009; Rosenberg and van Hout, 2013).

Therefore, in recent years, there has been a push towards automated sleep stage classification, enabled by advances in machine learning (Tsinalis et al., 2016; Supratak et al., 2017; Korkalainen et al., 2019; Mousavi et al., 2019; Phan et al., 2019; Seo et al., 2020; Guillot and Thorey, 2021). This development is further driven by the rise of new sleep monitoring technologies including wearable EEG. Wearable EEG facilitates the collection of more, longitudinal data that can be scored in a cost-efficient manner using automated sleep staging (Singh et al., 2015). Since wearable EEG usually uses less channels and potentially other electrode locations than the AASM standard, manual scoring is not always possible, while automated sleep stagers can be specifically trained on these channels (Finan et al., 2016; Garcia-Molina et al., 2018; Arnal et al., 2020). In particular, the use of deep neural network learning methods for automated sleep staging has yielded promising results, showing high agreement with manual scoring, similar or even superior to the inter-rater agreement between manual scorers (see, e.g., Fiorillo et al., 2019; Phan and Mikkelsen, 2022 for extensive reviews).

To reach expert-level performance using deep learning, hundreds of sleep recordings are typically required for model training (Biswal et al., 2018; Guillot and Thorey, 2021; Perslev et al., 2021). Databases such as the Montreal Archive of Sleep Studies (MASS; O’Reilly et al., 2014), the National Sleep Research Resource (NSRR; Zhang et al., 2018), and PhysioNet (Goldberger et al., 2000), together comprise thousands of sleep recordings and enable large scale training. However, training on publicly available data and subsequently employing the model on novel data of interest can be problematic when mismatches exist between source and target datasets, for example, with respect to population characteristics, or sensors.

Population mismatches can be present because population cohorts of public datasets generally contain either examples of healthy subjects, or mainly patients with common sleep disorders as obstructive sleep apnea (OSA) and insomnia. Patients with these sleep disorders can differ from healthy sleepers in sleep characteristics such as increased sleep fragmentation, and longer wake before sleep and after sleep onset (Mannarino et al., 2012; Baglioni et al., 2014). Many sleep disorders are much less prevalent, making data availability for training less abundant. Importantly, these sleep disorders can exhibit specific pathophysiological characteristics which are not learned by the model. The effects of such a population mismatch may, for example, appear in patients with REM sleep behavioral disorder (RBD). RBD patients are generally older, and the disorder is characterized by the absence of muscle atonia during REM sleep, causing dream-enacting behavior (Boeve et al., 2007; Sateia, 2014). Potentially, these characteristics contribute to the underperformance of automated sleep staging in RBD to healthy subjects and other sleep disorders, especially in REM sleep classification (Andreotti et al., 2018; Cooray et al., 2019).

Channel mismatches between the publicly available dataset and the novel dataset are the result when the electrode location of interest is not included in the publicly available data. For example, increasing interest in (prolonged) in-home sleep monitoring has led to the development of less obtrusive sleep monitoring technologies, including wearable EEG using dry frontopolar electrodes (Finan et al., 2016; Garcia-Molina et al., 2018; Arnal et al., 2020). The frontopolar location is not included in the majority of public available datasets, since it is not covered by AASM standards (Troester et al., 2023). Furthermore, compared to the standard wet electrodes in a PSG montage, dry electrodes in wearable systems result in lower signal quality and slightly different EEG signal information (Lopez-Gordo et al., 2014).

There are three different training strategies to employ an automated sleep stager on novel data, the new target dataset. In “pre-training”, the model is only trained on the original source dataset, often public data (Guillot and Thorey, 2021). Hence, sleep staging performance in the target dataset is susceptible to the presence of data mismatches (Phan et al., 2019; Phan et al., 2019). On the other hand, “training-from-scratch” can be used to train a new model only on the target dataset (Biswal et al., 2018; Perslev et al., 2021). However, data availability in the target dataset can be too limited for sufficient training of a deep learning model. For instance, the prevalence of specific sleep disorders such as RBD is low, and the validation of new EEG monitoring technologies is often limited to a minimal number of healthy subjects (Finan et al., 2016; Mikkelsen et al., 2017; Bresch et al., 2018; Sterr et al., 2018; Arnal et al., 2020). Recently, “fine-tuning” has been proposed as a solution to overcome data mismatches and limited data availability. In this form of transfer learning, the model is first pre-trained on the source dataset and further fine-tuned on the (smaller) target dataset to learn its specific characteristics (Pan and Yang, 2010). Although transfer learning for automated sleep staging shows promising results, performance improvement is limited (Andreotti et al., 2018; He et al., 2023). Also, it remains unknown how fine-tuning can best be implemented, since ideal settings seem specific to the deep learning architecture (Phan et al., 2019).

While each of the strategies (pre-training, training-from-scratch, and fine-tuning) has been used for training automated sleep stagers, no studies have been performed to systematically assess which method is favorable. The aim of this study was to evaluate which training strategy is preferred in the presence of data mismatches and limited data availability. For each strategy, we analyzed the performance on the combination of three age-matched populations (healthy subjects vs. OSA vs. insomnia) and one population with limited data availability (RBD), with two sets of EEG channels (F3-M2 vs. F3-F4). By comparing all strategies, populations, and channels in a systematic way, we could study the isolated effects of all these parameters. The TinySleepNet (Supratak and Guo, 2020), a previously published deep learning model for single-channel EEG, was used as automated sleep stage model. Both EEG channels and the automated model were chosen for their potential implementation in wearable EEG systems.

## 2 Materials and methods

### 2.1 Data

#### 2.1.1 Databases

The sleep-disordered participants were sampled from the PSG recordings of the Sleep and Obstructive Sleep Apnea Measuring with Non-Invasive Applications (SOMNIA) cohort recorded before January 2021 (van Gilst et al., 2019). All data was acquired at the Kempenhaeghe Center for Sleep Medicine (Heeze, the Netherlands) among individuals scheduled for an overnight PSG as part of the standard clinical routine. Trained clinicians manually scored the PSG recordings in accordance with AASM standards (Berry et al., 2017). The primary sleep diagnosis was coded according to the criteria specified in the International Classification of Sleep Disorders version 3 (ICSD-3).

The sleep recordings of healthy participants were obtained from the Healthbed database, which includes healthy adults without any known medical, psychiatric, or sleep disorders, recruited for an overnight PSG at Kempenhaeghe Center for Sleep Medicine (Heeze, the Netherlands) using the same setup as in the SOMNIA protocol (van Meulen et al., 2023).

The SOMNIA and Healthbed studies adhere to the guidelines of the Declaration of Helsinki, Good Clinical Practice, and current legal requirements. Both data collection studies were reviewed by the Maxima Medical Center medical ethical committee (Veldhoven, the Netherlands, reported under N16.074 and W17.128). The data analysis protocol was approved by the medical ethical committee of Kempenhaeghe Center for Sleep Medicine and the Philips Research Internal Committee for Biomedical Experiments.

#### 2.1.2 Source and target datasets

**FIGURE 1:**
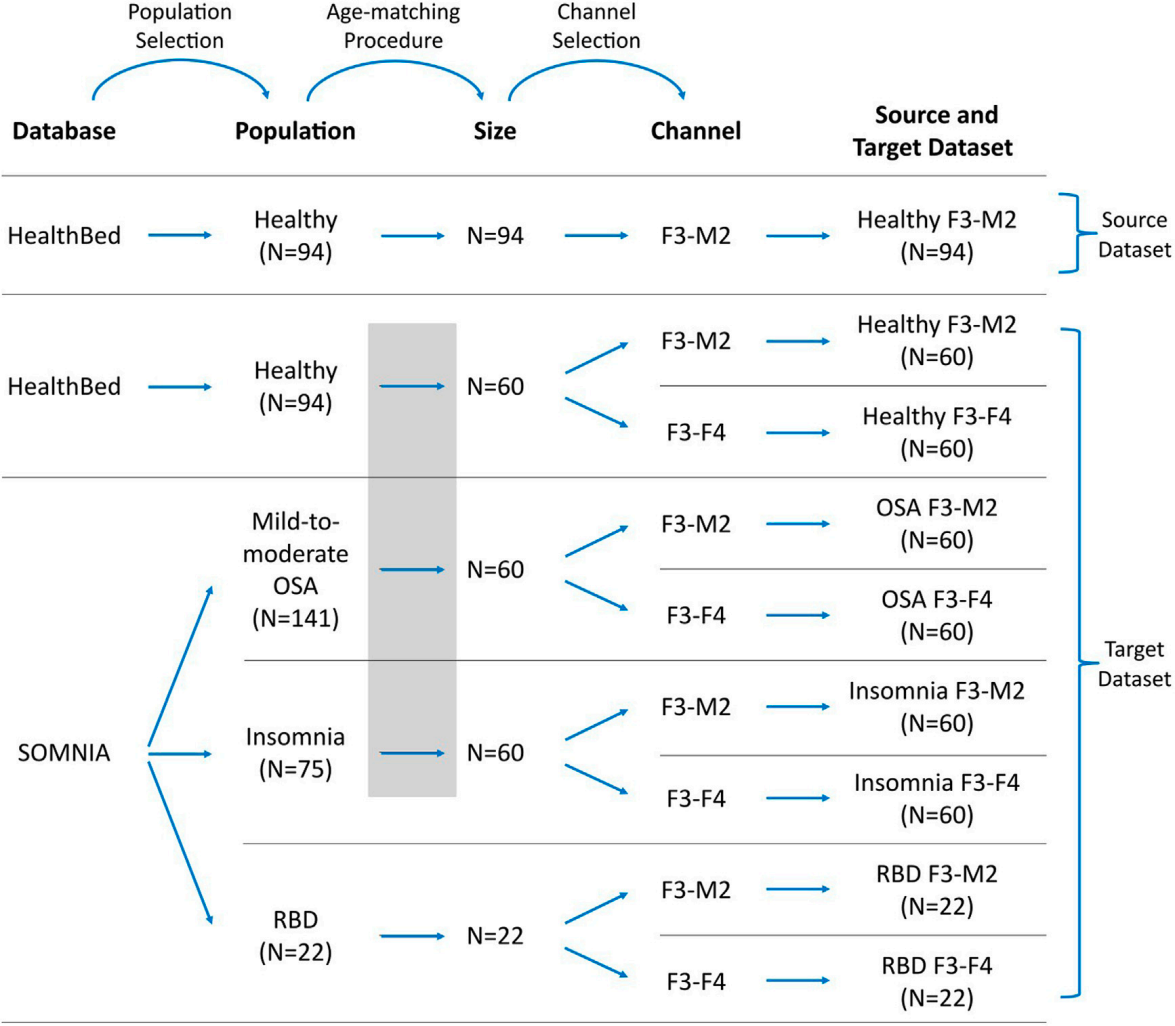
Workflow for defining the datasets. Datasets were selected from the Healthbed (van Meulen et al., 2023) and SOMNIA (van Gilst et al., 2019) databases. Population selection, an age-matching procedure, and channel selection resulted in one source dataset and eight target datasets. In target datasets, data availability can be limited, and/or can differ in characteristics to the source dataset due to population and channel mismatches. Grey box indicates the age-matching procedure for the healthy, OSA and insomnia target datasets.

A visual representation of how source and target datasets were defined, can be found in Figure 1.

We restricted our selection of source and target datasets to sleep recordings obtained from a single sleep center, thereby mitigating potential mismatches associated with inter-center variations, such as differences in sleep score training and in the PSG setup. For the source dataset, 94 sleep recordings of the F3-M2 EEG channel of the Healthbed database were used. These were all sleep recordings that were available from the Healthbed database.

For the target dataset the following selection procedure was used. We first included all 94 available Healthbed recordings. From the SOMNIA database we first included all patients with idiopathic, psychophysiological and/or chronic insomnia disorder, patients with mild-to-moderate OSA (apnea-hypopnea index (AHI) between 5 and 30, and patients with RBD. Patients with other diagnosed sleep disorder comorbidities were excluded. Since ageing is associated with an increasing variability, which can lower the generalization and thus can lower sleep staging performance (Guillot and Thorey, 2021), an age-matching algorithm was used to find the optimal grouping for the subjects of the healthy, OSA, and insomnia populations. Optimal grouping was defined as maximal subject inclusion and minimal age variance between the datasets, while keeping the age difference ≤5 years for each match. Age-matching resulted in 60 age-matched healthy, OSA, and insomnia subjects. In contrast, for the RBD target datasets no age-matching was performed. All 22 available subjects with RBD and without any other known sleep comorbidities, were included. The RBD datasets were defined as having limited data availability since, generally, a dataset size of 22 subjects is too small for sufficient training of a deep learning model including cross-validation.

Of each population, two target datasets were created comprising the F3-M2 and the F3-F4 EEG channel, resulting in a total of eight target datasets: healthy F3-M2, healthy F3-F4, OSA F3-M2, OSA F3-F4, insomnia F3-M2, insomnia F3-F4, RBD F3-M2, and RBD F3-F4.

#### 2.1.3 Data preprocessing

A 5th order Butterworth bandpass filter between .2 and 49 Hz, and a 50 Hz notch filter were applied to the raw data to select the frequency range of interest and remove power line interference for all recordings. Afterwards, data was down sampled from 512 to 100 Hz.

### 2.2 Deep learning model

We used the TinySleepNet deep learning model (Supratak and Guo, 2020) for 5-stage (Wake/N1/N2/N3/REM) automated sleep staging. TinySleepNet is a computationally efficient version of the DeepSleepNet (Supratak et al., 2017). The model has shown similar or superior performance to inter-rater agreement in manual scoring and to similar automated models (Supratak et al., 2017; Korkalainen et al., 2019; Mousavi et al., 2019). Notably, using the training-from-scratch strategy, model performance has been evaluated on seven public datasets and two electrode derivations (F4-EOG/C4-EOG and Fpz-Cz). The model has shown good generalizability with performances ranging between κ = .77 and κ = .82 (Supratak and Guo, 2020). The model contains 1.3 M parameters and can process (raw) single-channel EEG data. The representational learning component has four consecutive convolutional neural network (CNN) layers interleaved with two max-pooling and drop-out layers. The sequential learning component consists of a single, unidirectional long short-term memory layer (LSTM) and a drop-out layer. For further details we refer to the original work (Supratak and Guo, 2020).

### 2.3 Training strategies

We tested three different training strategies; pre-training, training-from-scratch and fine-tuning, which are described in detail below. For each training strategy, a 10-fold cross-validation was employed, allowing to evaluate the performance of the model on all subjects in the different datasets. In each iteration, 80% was used as training, 10% as validation, and 10% as test set, except for fine-tuning, where the number of subjects used for training was lowered (further specified in the corresponding section). Hence, the same subject was never included in more than one set at the same time.

The best model of each cross-validation iteration was selected based on the highest accuracy and weighted average of the F1-score in the validation set. Each model was trained for a maximum of 200 iterations with early stopping if no performance improvement in the validation set was observed in the next 50 training iterations.

#### 2.3.1 Pre-training

For pre-training, the TinySleepNet model was trained on all 94 F3-M2 EEG recordings of the healthy subjects (the source dataset). The model performance was then tested on each of the target datasets.

#### 2.3.2 Training-from-scratch

For training-from-scratch, the TinySleepNet was trained on subjects from the target dataset, while performance was also tested on subjects from the target dataset. As for each strategy, the set split was performed within the target dataset at subject level, hence a given subject was part of either the training set, the validation set, or the hold-out test set. The above described 10-fold cross validation procedure was used to ensure each subject was represented once in the test set. This procedure was repeated for each of the eight target datasets.

#### 2.3.3 Fine-tuning

For fine-tuning, the learning rate was lowered from 1e^−4^ to 1e^−5^ and only initial weights were loaded, without making any CNN layers non-trainable. These settings were derived from optimization experiments on separate Healthbed and SOMNIA data (i.e., not further used in the study).

Once fine-tuning parameters were optimized, the TinySleepNet model was first pre-trained on the source dataset. Afterwards a fine-tuning step was applied with a subset of the data from the target dataset. This procedure was repeated for each of the eight target datasets. The fine-tuning method was only tested in the sleep-disordered datasets (OSA, insomnia, RBD) and not on the healthy datasets, since fine-tuning a model on data on which the model was also pre-trained would be methodologically inappropriate.

For the OSA and insomnia datasets, additional training data of size n = 24 was used for fine-tuning, corresponding with 40% of the total target dataset, instead of the 80% using in pre-training and training-from-scratch. Since data availability was limited in RBD, additional training data of size n = 18 was used for fine-tuning, the maximum amount available.

To study how much additional training data from the target dataset is required for sufficient fine-tuning, we afterwards investigated three different conditions, referred to as fine-tuning l_3_, fine-tuning l_2_, and fine-tuning l_3_. Fine-tuning l_3_ corresponds with the dataset sizes described above, while additional training data was lowered to n = 12 in fine-tuning l_2_, and to n = 6 in fine-tuning l_1_. The absolute values and percentages used in fine-tuning are further specified in Table 1.

**TABLE 1:** Distributions of the training, validation, and test sets in fine-tuning l_1_, l_2_, and l_3_. Before fine-tuning, the model was first pre-trained on the source data. While train/validation/test set distributions were 80%/10%/10% in pre-training and training-from-scratch, the train data in fine-tuning systematically lowered. Set sizes of fine-tuning l_3_ have been used in analyses where the training strategy was compared to pre-training and training-from-scratch. Distributions in absolute subject numbers with percentages in parentheses.

Name	Train/validation/test set distributions in OSA and insomnia datasets (N = 60)	Train/validation/test set distribution for RBD datasets (N = 22)
Fine-tuning l_1_	6/6/6 (10%/10%/10%)	6/2/2 (30%/10%/10%)
Fine-tuning l_2_	12/6/6 (20%/10%/10%)	12/2/2 (55%/10%/10%)
Fine-tuning l_3_	24/6/6 (40%/10%/10%)	18/2/2 (80%/10%/10%)

### 2.4 Statistics

All performances were analyzed using Cohen’s Kappa coefficient of agreement (*κ*; Cohen, 1960) to compare the results of automatic scoring versus manual scoring on an epoch-per-epoch basis, for all stages (Wake, N1, N2, N3 and REM). In line with the general interpretation of the coefficient of agreement, κ ≥ .6 was defined as threshold of substantial performance (Landis and Koch, 1977). For each target dataset in each training strategy, sleep stage specific accuracy was given via confusion matrices in the Supplementary Material.

Performance differences between the training strategies (pre-training, training-from-scratch, and fine-tuning) were evaluated using a within-subject design. Considering the large sample size for the age-matched datasets, and no violation of normality and equality of variance in the RBD datasets, parametric tests were performed. Specifically, we used repeated measures ANOVAs, followed by *post hoc* paired samples t-tests if significant, including a Bonferroni correction of α/3 for multiple testing. Performance differences between the three different amounts of data in fine-tuning (l_1_, l_2_, and l_3_) were analyzed with the same procedure.

To analyze population and channel differences, performances across applied methods were first aggregated by either population or channel. Performance was compared using a between-subject design and parametric tests, due to sufficient sample sizes. Specifically, for population, differences between the age-matched healthy, OSA and insomnia datasets were tested with a one-way ANOVAs, followed by *post hoc* independent t-tests if significant. A Bonferroni correction of α/3 was applied to the significance threshold. For the channels, differences between the F3-M2 and F3-F4 databases were studied using an independent *t*-test.

## 3 Results

An overview of the demographic information and the PSG-derived sleep statistics of the datasets can be found in Table 2.

**TABLE 2:** Demographic and sleep information of source and target datasets. Healthy datasets included healthy adults without any known medical, psychiatric, or sleep disorders. The sleep-disordered OSA, insomnia, and RBD datasets included adults without any other known sleep comorbidities.

	Healthy (n = 94)	Healthy (n = 60)	OSA (n = 60)	Insomnia (n = 60)	RBD (n = 22)
Dataset	Source	Target	Target	Target	Target
Age	35.9 ± 13.5	42.4 ± 11.3	44.1 ± 11.0	43.4 ± 12.9	65.3 ± 7.0
Age Range	[18–64]	[20–64]	[22–65]	[20–64]	[50–79]
Sex (m/f)	36/58	26/34	48/12	30/30	13/9
BMI (kg/m^2^)	24.3 ± 3.2	25.2 ± 3.8	28.1 ± 5.4	25.6 ± 4.1	26.1 ± 4.7
TST (min)	431.7 ± 49.9	418.4 ± 52.6	414.1 ± 67.2	391.5 ± 68.6	393.8 ± 69.6
SOL (min)	11.3 ± 12.2	11.3 ± 13.7	18.5 ± 22.8	22.9 ± 17.1	13.5 ± 7.2
WASO (min)	33.7 ± 25.8	41.8 ± 28.1	42.7 ± 29.2	46.4 ± 36.6	59.6 ± 28.6
SE	87.9% ± 9.1%	85.9% ± 10.1%	82.1% ± 14.7%	80.1% ± 12.3%	79.2% ± 12.7%
Sleep Distribution	12% Wake	14% Wake	16% Wake	19% Wake	19% Wake
	8% N1	8% N1	11% N1	10% N1	13% N1
	43% N2	43% N2	43% N2	42% N2	41% N2
	19% N3	18% N3	16% N3	15% N3	13% N3
	18% REM	18% REM	14% REM	14% REM	13% REM

BMI, body mass index; AHI, apnea-hypopnea index; TST, total sleeping time; SE, sleep efficiency; WASO, wake after sleep onset; SOL, sleep onset latency.

### 3.1 Training strategies

**FIGURE 2:**
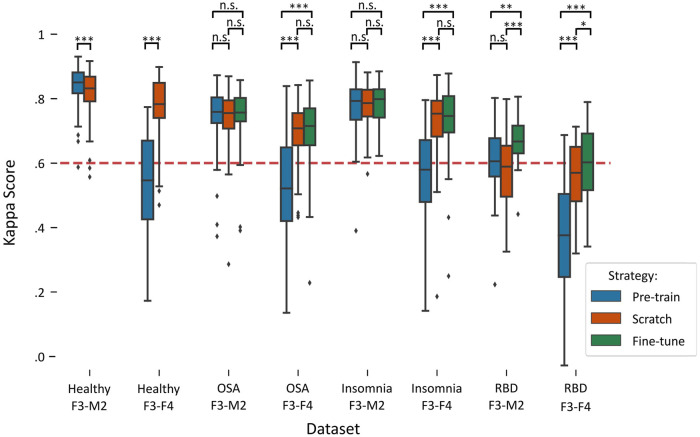
Boxplots of performances per training strategy in each target dataset. Red dashed line indicates the κ = .6 threshold of substantial agreement. Stars denote *p*-value of the test, where *, **, and *** denote *p* < .017, *p* < .01, and *p* < .001, respectively, while n.s. denotes “not significant” or *p* > .017. Training-from-scratch is abbreviated to “scratch”.

Figure 2 illustrates a boxplot of the performances on each target dataset using the three training strategies: pre-training, training-from-scratch, and fine-tuning. A detailed overview of the statistical differences between each training strategy can be found in Table 3.

**TABLE 3:** Performances of each training strategy and statistical differences. For each dataset, average Kappa agreement and the percentage of subjects above the threshold of substantial agreement (*κ* > .6) is given, followed by the group statistic and *post hoc* analysis between training strategies if significant. For the healthy datasets, no group statistic is shown since comparison is between two training strategies. Training-from-scratch is abbreviated to “scratch”.

Dataset	N	Method	Kappa Mean ± SD	Above substantial agreement (%)	Repeated measures ANOVA: *p*-value, effect size	Post-hoc comparison between	Paired samples t-test: *p*-value, effect size
Healthy F3-M2	60	Pre-train	κ = .84 ± .06	98%		Pre-train & Scratch	*p* < .001, *d* = .27
		Scratch	κ = .82 ± .08	97%			
Healthy F3-F4	60	Pre-train	κ = .53 ± .16	35%		Pre-train & Scratch	*p* < .001, *d* = 1.87
		Scratch	κ = .77 ± .09	93%			
OSA F3-M2	60	Pre-train	κ = .74 ± .10	93%	*p* = .26, η_p_^2^ = .002		
		Scratch	κ = .74 ± .09	95%			
		Fine-tune	κ = .75 ± .09	95%			
OSA F3-F4	60	Pre-train	κ = .53 ± .17	40%	*p* < .001, η_p_^2^ = .29	Pre-train & Scratch	*p* < .001, *d* = 1.26
		Scratch	κ = .70 ± .09	87%		Pre-train & Fine-tune	*p* < .001, *d* = 1.21
		Fine-tune	κ = .70 ± .11	83%		Scratch & Fine-tune	*p* = .84, *d* = .02
Insomnia F3-M2	60	Pre-train	κ = .77 ± .09	98%	*p* = .27, η_p_^2^ = .003		
		Scratch	κ = .77 ± .07	98%			
		Fine-tune	κ = .78 ± .07	100%			
Insomnia F3-F4	60	Pre-train	κ = .56 ± .13	43%	*p* < .001, η_p_^2^ = .23	Pre-train & Scratch	*p* < .001, *d* = 1.39
		Scratch	κ = .73 ± .10	93%		Pre-train & Fine-tune	*p* < .001, *d* = 1.42
		Fine-tune	κ = .73 ± .11	92%		Scratch & Fine-tune	*p* = .53, *d* = .04
RBD F3-M2	22	Pre-train	κ = .60 ± .13	50%	*p* = .002, η_p_^2^ = .11	Pre-train & Scratch	*p* = .43, *d* = .18
		Scratch	κ = .58 ± .12	45%		Pre-train & Fine-tune	*p* = .001, *d* = .65
		Fine-tune	κ = .67 ± .08	91%		Scratch & Fine-tune	*p* < .001, *d* = .89
RBD F3-F4	22	Pre-train	κ = .37 ± .18	14%	*p* < .001, η_p_^2^ = .31	Pre-train & Scratch	*p* < .001, *d* = 1.06
		Scratch	κ = .54 ± .13	36%		Pre-train & Fine-tune	*p* < .001, *d* = 1.51
		Fine-tune	κ = .60 ± .11	55%		Scratch & Fine-tune	*p* = .01, *d* = .51

In the healthy F3-M2 dataset, both training strategies achieved performance higher than κ = .6, the threshold of substantial agreement according to the general interpretation of the statistic (Landis and Koch, 1977). Pre-training performance (*κ* = .84 ± .06) was slightly but significantly higher than training-from-scratch (*κ* = .82 ± .08). In contrast, for the healthy F3-F4 dataset, pre-training (*κ* = .53 ± .16) achieved an average performance lower than substantial agreement threshold, while training-from-scratch (*κ* = .77 ± .09) obtained significantly higher performance.

In the OSA F3-M2 dataset, no difference between pre-training (*κ* = .74 ± .10), training-from-scratch (*κ* = .74 ± .09), and fine-tuning (*κ* = .75 ± .09) was observed. In the OSA F3-F4 dataset, pre-training (*κ* = .53 ± .17) on average performed under the threshold of substantial agreement, with significantly lower performance compared to training-from-scratch (*κ* = .70 ± .09) and fine-tuning (*κ* = .70 ± .11).

In the insomnia F3-M2 dataset, no difference between pre-training (*κ* = .77 ± .09), training-from-scratch (*κ* = .77 ± .07), and fine-tuning (*κ* = .78 ± .07) was observed. In the insomnia F3-F4 dataset, pre-training (*κ* = .56 ± .13) on average performed under the threshold of substantial agreement, with significantly lower performance compared to training-from-scratch (*κ* = .73 ± .10) and fine-tuning (*κ* = .73 ± .11).

In the RBD F3-M2 dataset, fine-tuning (*κ* = .67 ± .08) significantly outperformed pre-training (*κ* = .60 ± .13) and training-from-scratch (*κ* = .57 ± .12), with training-from-scratch on average performing under the threshold of substantial agreement. Similarly, in the RBD F3-F4 dataset, performance was significantly higher in fine-tuning (*κ* = .60 ± .11) than in pre-training (*κ* = .37 ± .18) and training-from-scratch (*κ* = .54 ± .13). Here, training-from-scratch significantly outperformed pre-training, although on average both performed under the threshold of substantial agreement.

Sleep stage specific performance results for each target dataset are detailed in Supplementary Figures S1–S4. In general, a decrease in performance across all sleep stages was observed in target datasets with lower Kappa scores. In addition, two notable observations can be made. First, in all RBD datasets, a relatively high degree of missed REM sleep is demonstrated, with REM recall ranging between 26.1% and 61.6% accuracy. Second, in the pre-training strategy on all F3-F4 dataset, 46.0%–61.4% of the N3 epochs were incorrectly classified as N2 sleep.

### 3.2 Population differences

In pre-training, no significant differences between the three populations were observed using a one-way ANOVA, *p* = .09, η_p_^2^ = .01.

In training-from-scratch, significant differences between the three populations were found, *p* < .001, η_p_^2^ = .11. Post-hoc independent t-tests showed higher performance in the aggregated healthy datasets (*κ* = .80 ± .09) than in OSA (*κ* = .72 ± .09), *p* < .001, *d* = .86, and insomnia (*κ* = .75 ± .09), *p* < .001, *d* = .50. Also, outperformance of insomnia compared to OSA was shown, *p* = .005, *d* = .37.

In fine-tuning, higher performance in insomnia (*κ* = .76 ± .09) than in OSA (*κ* = .72 ± .10) was observed, *p* = .007, *d* = .35.

### 3.3 Channel differences

To study the effect of channel on performance, results of the three training strategies (pre-training, training-from-scratch and fine-tuning) in each age-matched population (healthy, OSA, and insomnia) were grouped by either the F3-M2 or the F3-F4 electrode channel. An independent *t*-test showed significantly higher performance using the F3-M2 channel (*κ* = .78 ± .09) than using the F3-F4 channel (*κ* = .66 ± .16), *p* < .001, *d* = .96.

### 3.4 Effect of set size in fine-tuning

**FIGURE 3:**
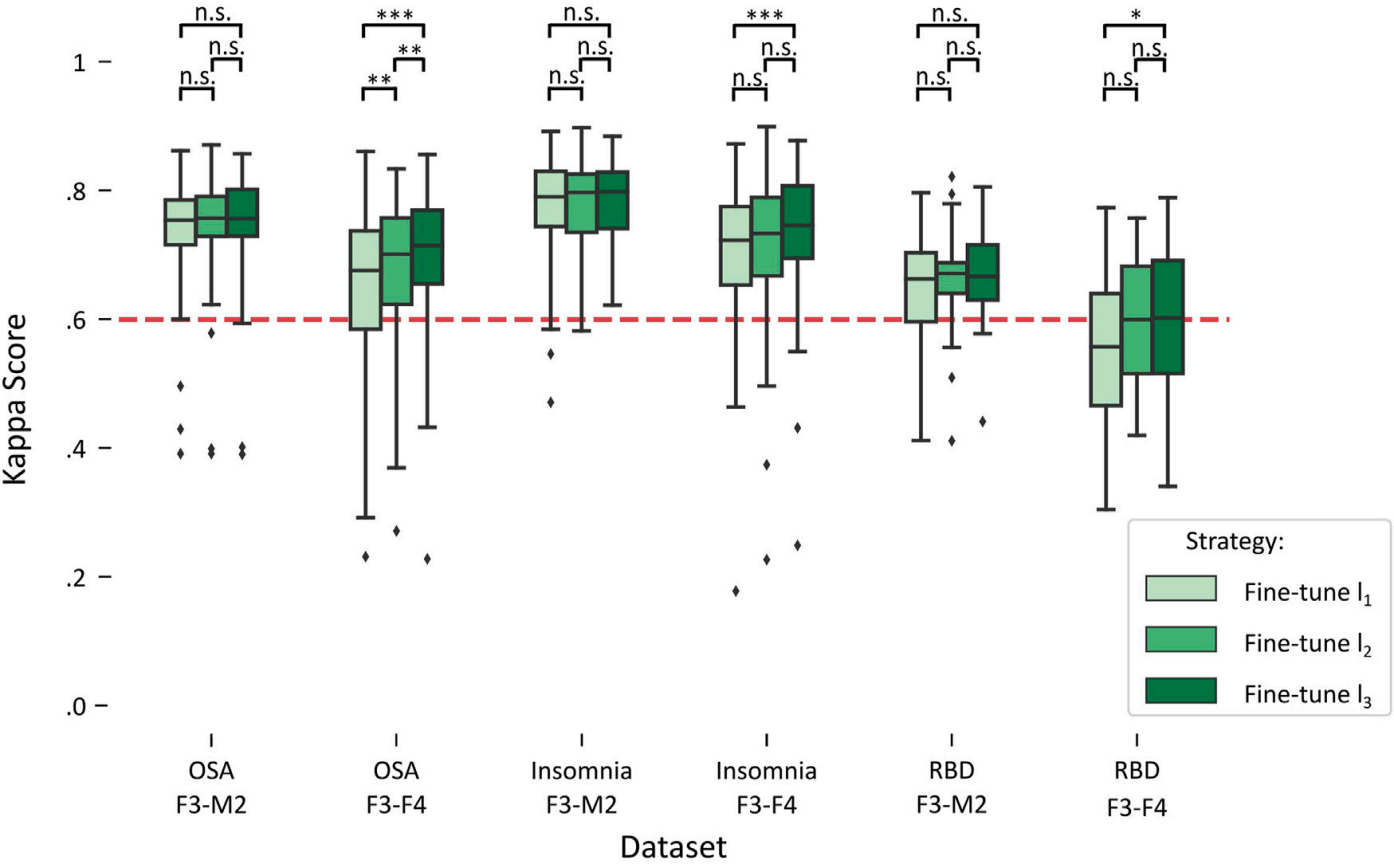
Boxplots of performances for fine-tune l_1_, l_2_, and l_3_ in each target dataset. Red dashed line indicates the κ = .6 threshold of substantial agreement. Stars denote *p*-value of the test, where *, **, and *** denote *p* < .017, *p* < .01, and *p* < .001, respectively, while n.s. denotes “not significant” or *p* > .017.

Figure 3 illustrates the impact on performance when including more subjects from the target dataset for fine-tuning, referred to as fine-tuning l_1_ (lowest amount), fine-tuning l_2_ (medium amount), and fine-tuning l_3_ (highest amount). A detailed overview of the statistical differences can be found in Table 4.

**TABLE 4:** Fine-tune l_1_, l_2_, and l_3_ performances and statistical differences. For each dataset, average Kappa agreement and the percentage of subjects above the threshold of substantial agreement (*κ* > .6) is given, followed by the group statistic and *post hoc* analysis between fine-tune l_1_, l_2_, and l_3_ if significant.

Dataset	N	Fine-tune size	Kappa Mean ± SD	Substantial agreement (%)	Repeated measures ANOVA: *p*-value, effect size	Post-hoc comparison between	Paired samples t-test: *p*-value, effect size
OSA F3-M2	60	l_1_	κ = .74 ± .09	95%	*p* = .17, η_p_^2^ = .003		
		l_2_	κ = .74 ± .09	95%			
		l_3_	κ = .75 ± .09	95%			
OSA F3-F4	60	l_1_	κ = .65 ± .13	73%	*p* < .001, η_p_^2^ = .03	l_1_ & l_2_	*p* = .003, *d* = .20
		l_2_	κ = .67 ± .12	82%		l_1_ & l_3_	*p* < .001, *d* = .39
		l_3_	κ = .70 ± .11	83%		l_2_ & l_3_	*p* = .007, *d* = .20
Insomnia F3-M2	60	l_1_	κ = .77 ± .09	95%	*p* = .05, η_p_^2^ = .005		
		l_2_	κ = .77 ± .08	97%			
		l_3_	κ = .78 ± .07	100%			
Insomnia F3-F4	60	l_1_	κ = .70 ± .11	85%	*p* < .001, η_p_^2^ = .01	l_1_ & l_2_	*p* = .20, *d* = .06
		l_2_	κ = .71 ± .12	85%		l_1_ & l_3_	*p* < .001, *d* = .28
		l_3_	κ = .73 ± .11	92%		l_2_ & l_3_	*p* < .001, *d* = .21
RBD F3-M2	22	l_1_	κ = .65 ± .09	73%	*p* = .28, η_p_^2^ = .009		
		l_2_	κ = .66 ± .09	77%			
		l_3_	κ = .67 ± .08	91%			
RBD F3-F4	22	l_1_	κ = .56 ± .12	41%	*p* = .02, η_p_^2^ = .03	l_1_ & l_2_	*p* = .04, *d* = .39
		l_2_	κ = .60 ± .10	50%		l_1_ & l_3_	*p* = .01, *d* = .38
		l_3_	κ = .60 ± .11	55%		l_2_ & l_3_	*p* = .99, *d* = .00

For each of the datasets with the F3-M2 channel datasets (OSA F3-M2, insomnia F3-M2, and RBD F3-M2), a repeated measures ANOVA indicated no significant differences between l_1_, l_2_, and l_3_. In contrast, significant differences were observed for each dataset with the F3-F4 channel (OSA F3-F4, insomnia F3-F4, and RBD F3-F4). Notably, in OSA and insomnia F3-F4 datasets, average performance above substantial agreement is reached in l_1_. In RBD F3-F4, substantial agreement is realized in l_2_.

### 3.5 Fine-tuning dynamics

**FIGURE 4:**
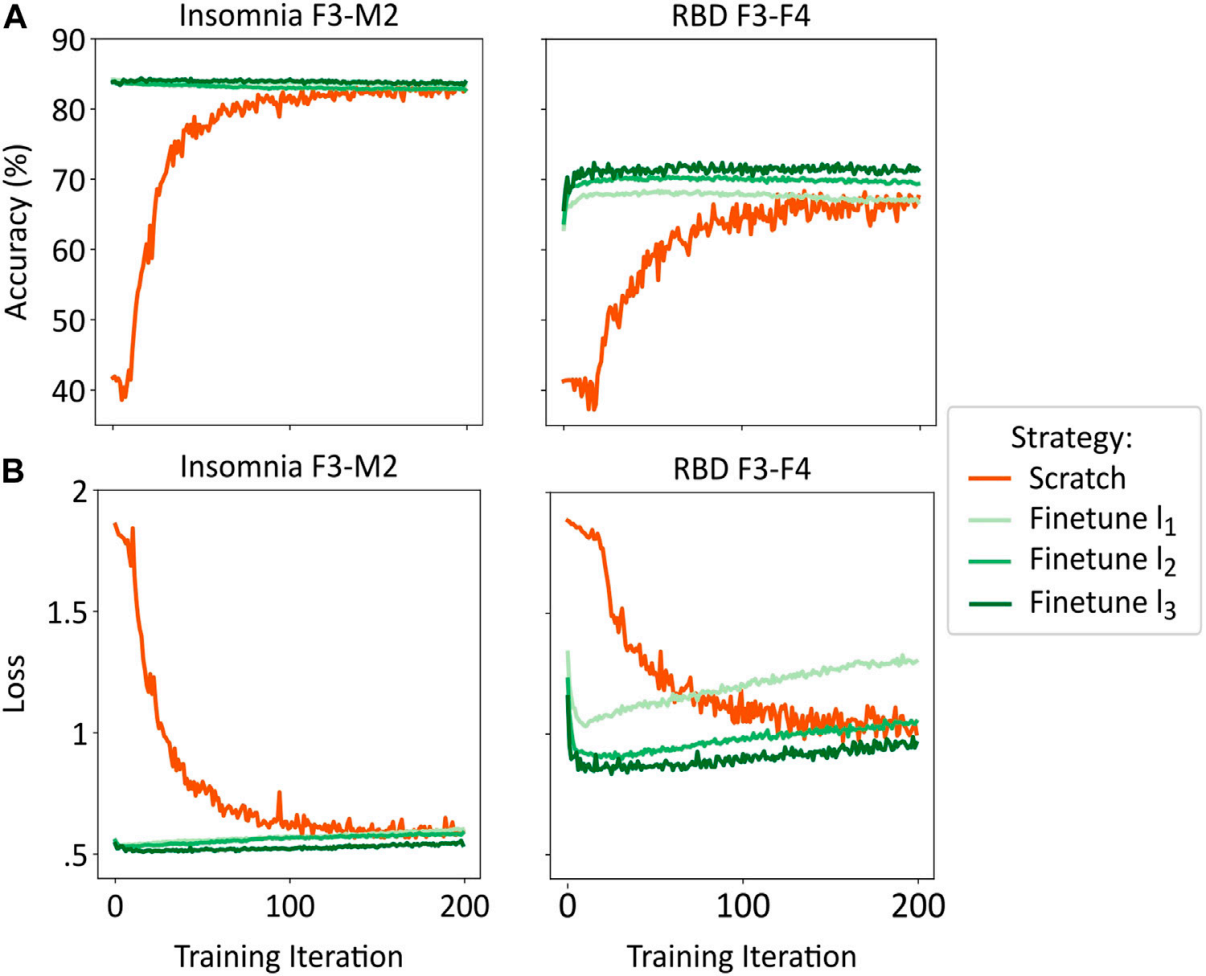
Examples of accuracy **(A)** and loss function **(B)** for training-from-scratch (abbreviated to “scratch”), fine-tuning l_1_, l_2_, and l_3_ for the best (insomnia F3-M2, left) and worst (RBD F3-F4, right) performing validation sets. Averaged across the ten models of the cross-validation.

The loss function for fine-tuning showed that optimal loss was achieved after 5 to 35 training iterations, depending on the target dataset and the number of subjects used for fine-tuning, after which overfitting occurred (see Figure 4A). A small number of subjects used for fine-tuning led to faster loss increase. In contrast, a logarithmic decrease in loss function was observed in training-from-scratch.

The model’s accuracy exhibited similar differences between the training-from-scratch and fine-tuning methods. While in training-from-scratch, accuracy followed a logarithmic increase, optimal accuracy in fine-tuning was reached early, stabilized, and potentially decreased slightly as training continued (see Figure 4B).

For fine-tuning, learning rate was set to 1e^−5^ and only initial weights were loaded, without making any CNN layers non-trainable. Higher learning rates and an increasing number of non-trainable CNN layers exhibited similar loss functions, albeit faster overfitting occurred. Also, with higher learning rates, accuracy was lower and plateaued earlier.

## 4 Discussion

In this study we evaluated three strategies (pre-training, training-from-scratch, fine-tuning) for training an automated single-channel EEG sleep staging model when mismatches between the source and target dataset are present. Each strategy was tested on a total of eight target datasets, comprising of healthy subjects and patients with OSA, insomnia, or RBD; combined with the F3-M2 and F3-F4 EEG channels.

### 4.1 Model performance

First of all, our results illustrate the strong performance of the TinySleepNet automated sleep staging model (Supratak and Guo, 2020). Our Healthbed dataset yielded slightly higher agreement (*κ* = .84 and κ = .82 in pre-trained and trained-from-scratch healthy F3-M2 datasets, respectively) compared to previously reported performances on the sleep-EDF (*κ* = .77–.80) and MASS (*κ* = .77–.82) datasets (Supratak and Guo, 2020). Other automated single-channel EEG models have shown similar performance for sleep-EDF dataset (*κ* = .81; Phan et al., 2019) and MASS dataset (*κ* = .78–82; Phan et al., 2019; Mousavi et al., 2019; Seo et al., 2020).

The average performance in the OSA (*κ* = .74) and insomnia (*κ* = .77) datasets on the F3-M2 channel were lower compared to healthy individuals, with medium to large effect sizes ranging between *d* = .35 and *d* = .86 (Cohen, 1960). Since datasets were age-matched, sampled from the same database, and other known sleep comorbidities were excluded, underperformance with respect to healthy subjects can potentially be attributed to the sleep characteristics of the disorders. An earlier study with frontal (wearable) single-channel EEG automated sleep staging reported underperformance specifically in the differentiation of N1 sleep due to a lack of measured occipital activity (Lucey et al., 2016). This can particularly affect performance in OSA and insomnia due to the increased sleep fragmentation. Another possible explanation is that the dataset sizes used for training in this study have been too small to sufficiently capture the heterogeneity of sleep within in these sleep-disordered populations.

In RBD, agreement levels of κ = .67 (F3-M2 channel) and κ = .60 (F3-F4 channel) were obtained using fine-tuning, an average increase of κ = .15 compared with pre-training and an average increase of κ = .08 compared with training-from-scratch. Despite reaching or exceeding substantial agreement according to the general interpretation of the Kappa statistic (Landis and Koch, 1977), performance still falls below that obtained for the other tested sleep disorders. Although, to the best of our knowledge, studies evaluating human inter-rater agreement for sleep scoring in RBD are lacking, relatively low agreement has been reported in presence of Parkinson’s disease (*κ* = .61; Danker-Hopfe et al., 2004), a neurodegenerative disease strongly associated with RBD (Schenck, Boeve and Mahowald, 2013). Automated sleep scoring performance in RBD was higher than previously described in a cohort of 22 RBD subjects, with performances of κ = .45 before and κ = .56 after subject-specific fine-tuning (i.e., fine-tuning on each patient’s first night PSG, tested on the second night; Andreotti et al., 2018).

### 4.2 Training strategies

Several conclusions can be drawn from the study regarding the preferred training strategy in presence of population mismatches. In the age-matched OSA F3-M2 and insomnia F3-M2 datasets, no difference in performance between the three training strategies was observed, implying it is inconsequential whether the model was trained on the healthy F3-M2 source dataset (pre-training), target dataset (training-from-scratch) or both (fine-tuning). These results suggest a similarity in data characteristics between the healthy source and the OSA and insomnia target when datasets are solely mismatched for these sleep disorders, without the presence of age-related population mismatches. Hence, sleep of OSA and insomnia patients is considered abnormal, and abnormal characteristics including increased sleep fragmentation are likely decreasing sleep staging performance. However, these are not characteristics that cause population mismatches and thus require targeted training.

In contrast, for the RBD F3-M2 dataset, differences between the training strategies were found. Underperformance in pre-training suggests specific characteristics in RBD that the model needs to train on, either caused by the older age of RBD patients and/or by sleep characteristics inherent to RBD. Previously reported lower agreement in RBD (*κ* = .54) in comparison to age-matched healthy subjects (*κ* = .73) indicate likely not all underperformance in RBD is age-related (Cooray et al., 2019). Notably, for each of the training strategies, the missed classification of REM sleep is especially problematic, but lower agreement in RBD is observed across all sleep stages (see Supplementary Figure S4). These findings are in line with earlier studies (Andreotti et al., 2018; Cooray et al., 2019). We hypothesize that the frontopolar EEG channel can capture the characteristic elevated muscle activity during REM sleep in RBD, complicating the correct classification of REM sleep when muscle tone is present. Additionally, the generally lower performance could be attributed to microstructural changes and decreased sleep stability (Christensen et al., 2016; Cesari et al., 2021) that are associated with RBD. However, future research on RBD sleep staging is needed for the further characterization of the lower performance. Underperformance in training-from-scratch can likely be explained because insufficient RBD data is available to obtain robust performance. This practical challenge can emerge given that RBD is a less prevalent sleep disorder. Hence, when differences in data characteristics are present and target data availability is limited, fine-tuning is the preferred training strategy. Medium to large effect sizes (*d* = .65–.87; Cohen, 1960) emphasize the strong advantages of fine-tuning for RBD data.

Results in the F3-F4 target datasets suggest that, when channel mismatches with the source dataset are present, pre-training (on the F3-M2 channel) is not sufficient. Training on the target is necessary through either training-from-scratch or fine-tuning. Notably, fine-tuning delivers similar classification performance to training-from-scratch, while requiring only half the amount of target data. The threshold of substantial agreement is already exceeded with fine-tuning on 12 (6 train +6 validation) target subjects in OSA and insomnia. Again, for the RBD F3-F4 dataset specifically, fine-tuning is preferred since the dataset is too small for training-from-scratch. Here, substantial agreement is reached with 14 (12 train +2 validation) target subjects.

### 4.3 Transfer learning

Making several layers of a model non-trainable is perceived as a common method for transfer learning (e.g., Shin et al., 2016; Tajbakhsh et al., 2016). However, for TinySleepNet, we achieved best performance by fine-tuning the full model, hence making no layers non-trainable and only loading in the weights of the pre-trained model. This irregularity is possibly explained by the low computational costs and straight-forward design of the model, making it more flexible to tune to new data characteristics. Furthermore, early stopping and a lowered learning rate (from 1e^−4^ to 1e^−5^) were necessary to prevent the model from overfitting on the target data, especially when larger mismatches with the source dataset were present and/or when target data availability was limited. Optimal model performance in fine-tuning was reached after 5 to 35 training iterations, while requiring at least 80 training iterations when using pre-training or training-from-scratch as training strategy, emphasizing the lower computational costs for transfer learning. Our findings are similar to observations for fine-tuning in channel mismatches using the SeqSleepNet model, where full fine-tuning of the model showed superior sleep staging performance for transferring from the C4-A1 channel in the MASS dataset to the Fpz-Cz channel in the sleep-EDF dataset (Phan et al., 2019). However, in the DeepSleepNet, an architecture more similar to the TinySleepNet, fine-tuning only the softmax layer for channel changes seems to yield highest performance (Phan et al., 2019). These results suggest fine-tuning is a model and mismatch specific process, and no one-size-fits-all strategy is (yet) known.

### 4.4 Single-channel (wearable) EEG

There is an ongoing debate on the preferred channel for single-channel EEG automated sleep staging, including the suggested use of Fpz-Cz, Pz-Oz (see, e.g., Supratak et al., 2017), and Fp1-Fp2 (Radha et al., 2014). In this study, we chose to include leads present in the PSG setup which are as close as possible to the frontopolar locations often used in wearable EEG. For the development of these technologies, it is valuable to understand how public PSG databases can be leveraged through pre-training and fine-tuning. Especially since validation of wearable (single-channel) EEG is often limited to small and homogeneous recordings of healthy subjects because of costs, time, and ethical regulations (Finan et al., 2016; Garcia-Molina et al., 2018; Arnal et al., 2020). Also, in contrast to similar models (Supratak et al., 2017; Korkalainen et al., 2019; Mousavi et al., 2019), TinySleepNet can potentially be implemented for real-time sleep stage classification in wearable EEG, due to the model’s architecture. With the fine-tuning approach used in this study, we have shown that such a sleep staging model can also be trained for less prevalent disorders with specific characteristics, such as RBD. Furthermore, the use of wearable EEG for prolonged monitoring and the collection of longitudinal data can enable new possibilities to develop personalized sleep staging models, wherein (subject-specific) fine-tuning can play a critical role (Andreotti et al., 2018; Phan et al., 2020).

Our results consistently show underperformance of the F3-F4 channel (*κ* = .66) when compared with the F3-M2 channel (*κ* = .78) with a large effect (*d* = .96; Cohen, 2013), possibly because the F3 and F4 locations share more signal characteristics of interest, which are subtracted when re-referencing the channels. Two studies have been performed in the sleep-disordered population using frontal channels in wearable EEG, both showing κ = .67 agreement levels (Lucey et al., 2016; Levendowski et al., 2017). It should be noted that also lower inter-rater reliability for manual scoring of the recordings is reported (*κ* = .69 in Garcia-Molina et al., 2019; κ = .70 in Levendowski et al., 2017; κ = .78 in Popovic et al., 2014), suggesting an upper limit to the sleep staging information extracted from wearable frontal electrodes.

Furthermore, it is imperative to acknowledge that sleep characteristics may exhibit variation across channels, and thus can cause channel mismatches. A prime example is the reduced N3 classification performance observed in the F3-F4 datasets when the model is pre-trained on F3-M2, in contrast to the results obtained with training-from-scratch or fine-tuning on F3-F4 (see Supplementary Figures S1–S4). These findings suggest a distinct manifestation of the characteristic N3 slow wave sleep in the two channels.

### 4.5 Limitations

Some limitations of the current study should be considered. First, while typically hundreds of sleep recordings are required to reach expert-level performance using deep learning (Biswal et al., 2018; Guillot and Thorey, 2021; Perslev et al., 2021), in the current study, 94 recordings for pre-training, and 60 recordings for training-from-scratch, have been used. Potentially, larger sample sizes can achieve better model training and higher performance, especially in the sleep-disordered populations which can be characterized by increased variability. However, the goal of the current study was to investigate different training strategies in the presence of data mismatches. Hence, only data from one sleep center was used, allowing to isolate population and channel mismatches, and avoiding potential additional mismatches from PSG setup differences or differences in manual sleep score training.

Second, TinySleepNet discerns from other related automated models for the potential implementation into wearable EEG due to its low computation costs and unidirectional LSTM, which allows for real-time classification. However, implementation here is only theorized, and should be tested in future research. Since the current study is limited to gold-standard EEG recordings only, it remains unknown how fine-tuning performance is affected when recordings from dry electrodes with higher signal-to-noise ratios are used as target.

Last, one should consider the generalizability of this study carefully. Although other single-channel EEG deep learning classification models (Tsinalis et al., 2016; Supratak et al., 2017; Korkalainen et al., 2019; Mousavi et al., 2019; Phan et al., 2019; Seo et al., 2020; Guillot and Thorey, 2021) have related architectures, their differences potentially result in model-specific transfer learning dynamics (Phan et al., 2019). Similarly, the preferred applied methods that have been investigated in this work are possibly specific to the database, available amount of data, and mismatches between the source and target datasets.

## 5 Conclusion

In this work, we have shown that the preferred training strategy for automated single-channel automated sleep stager depends on the presence of data mismatches, type of mismatch, and the availability of data. OSA and insomnia target datasets show no population mismatches when the model is pre-trained on healthy individuals. In contrast, RBD sleep recordings contain characteristics, either inherent to the pathology or age-related, which demand targeted model training. Targeted training is also needed when source and target datasets differences cause channel mismatches. In the presence of these data mismatches, fine-tuning can yield similar to superior performance than training-from-scratch, with a significantly reduced dataset size.

## Data Availability

The Healthbed (van Meulen et al., 2023) and SOMNIA (van Gilst et al., 2019) datasets, collected by Kempenhaeghe Center for Sleep Medicine. Further information on data availability can be obtained in the original works. The TinySleepNet model (Supratak and Guo, 2020) is available on https://github.com/akaraspt/tinysleepnet.
