# Supplementary material for: Deep transfer learning for automated single-lead EEG sleep staging with channel and population mismatches
Source: Front Physiol. 2024 Jan 5;14:1287342. doi: 10.3389/fphys.2023.1287342 (PMC10796543; doi:10.3389/fphys.2023.1287342)
Supplement: Supplementary file 1 [file Image1.pdf]

Supplementary Material

Deep Transfer Learning for Automated Single-Lead EEG Sleep Staging with Channel and Population Mismatches

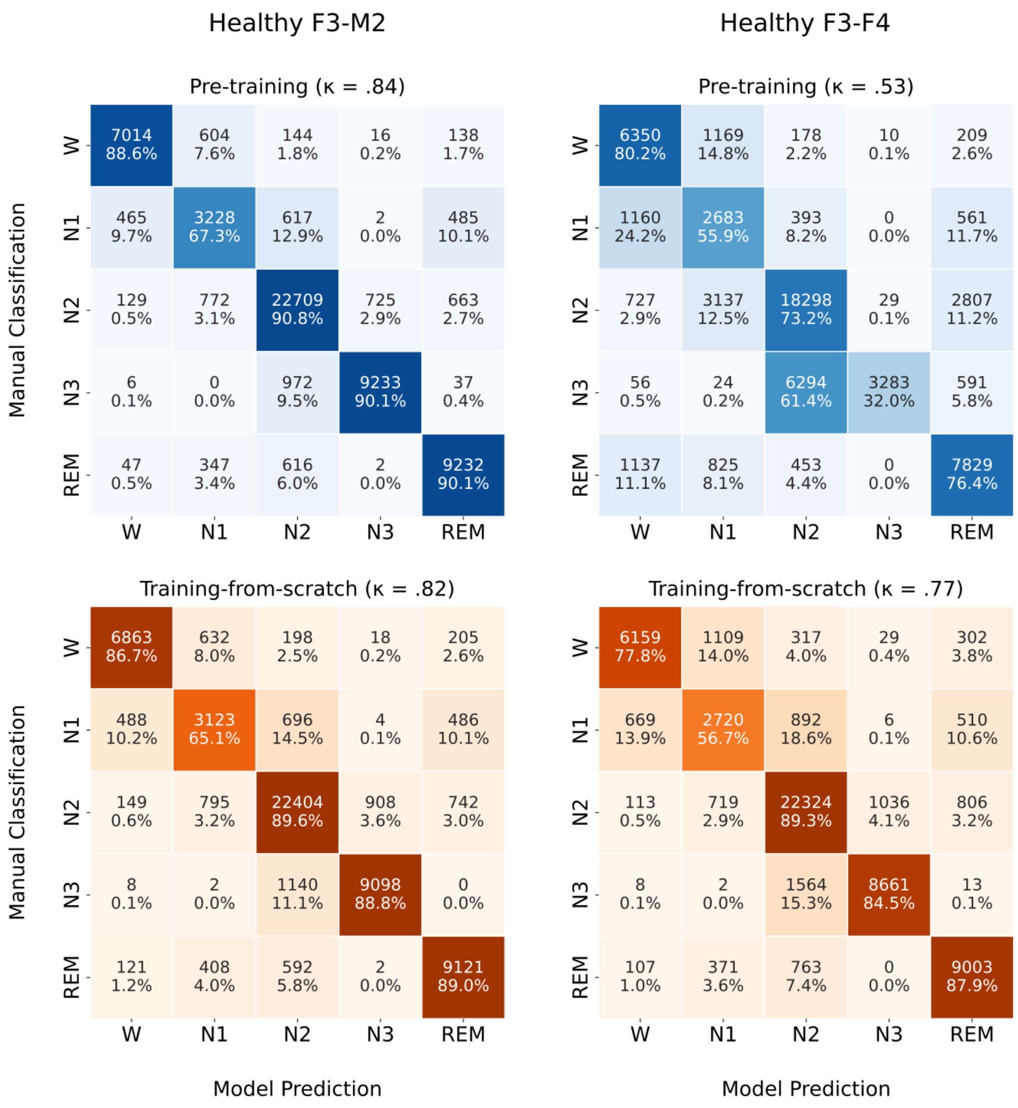

Supplementary Figure 1. Confusion matrices between manual and model classification for the training strategies in the Healthy F3-M2 (left) and F3-F4 (right) datasets. Absolute number of epochs and percentage accuracy are given. Darker hue indicates higher accuracy.

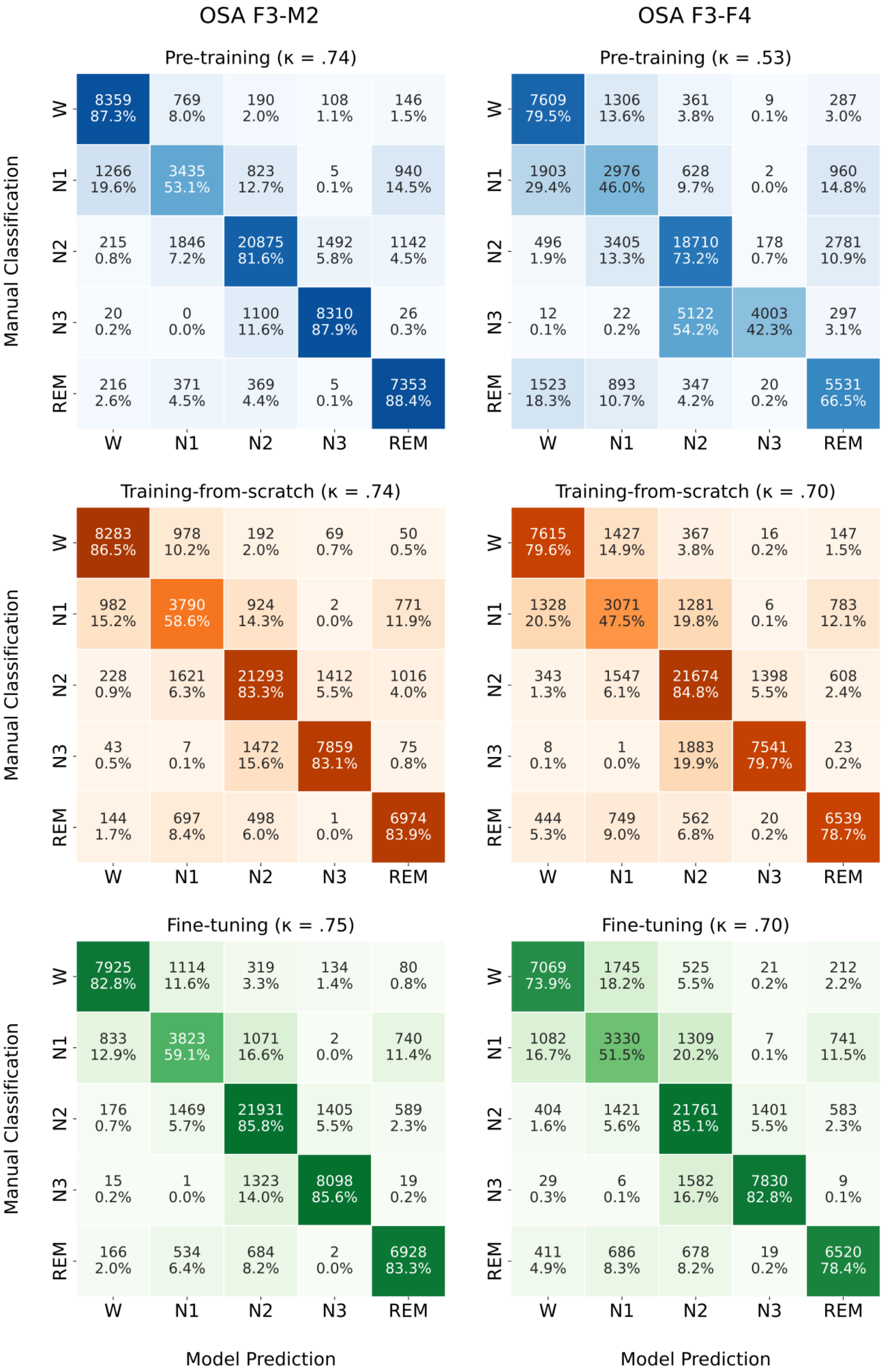

Supplementary Figure 2. Confusion matrices between manual and model classification for the training strategies in the OSA F3-M2 (left) and F3-F4 (right) datasets. Absolute number of epochs and percentage accuracy are given. Darker hue indicates higher accuracy.

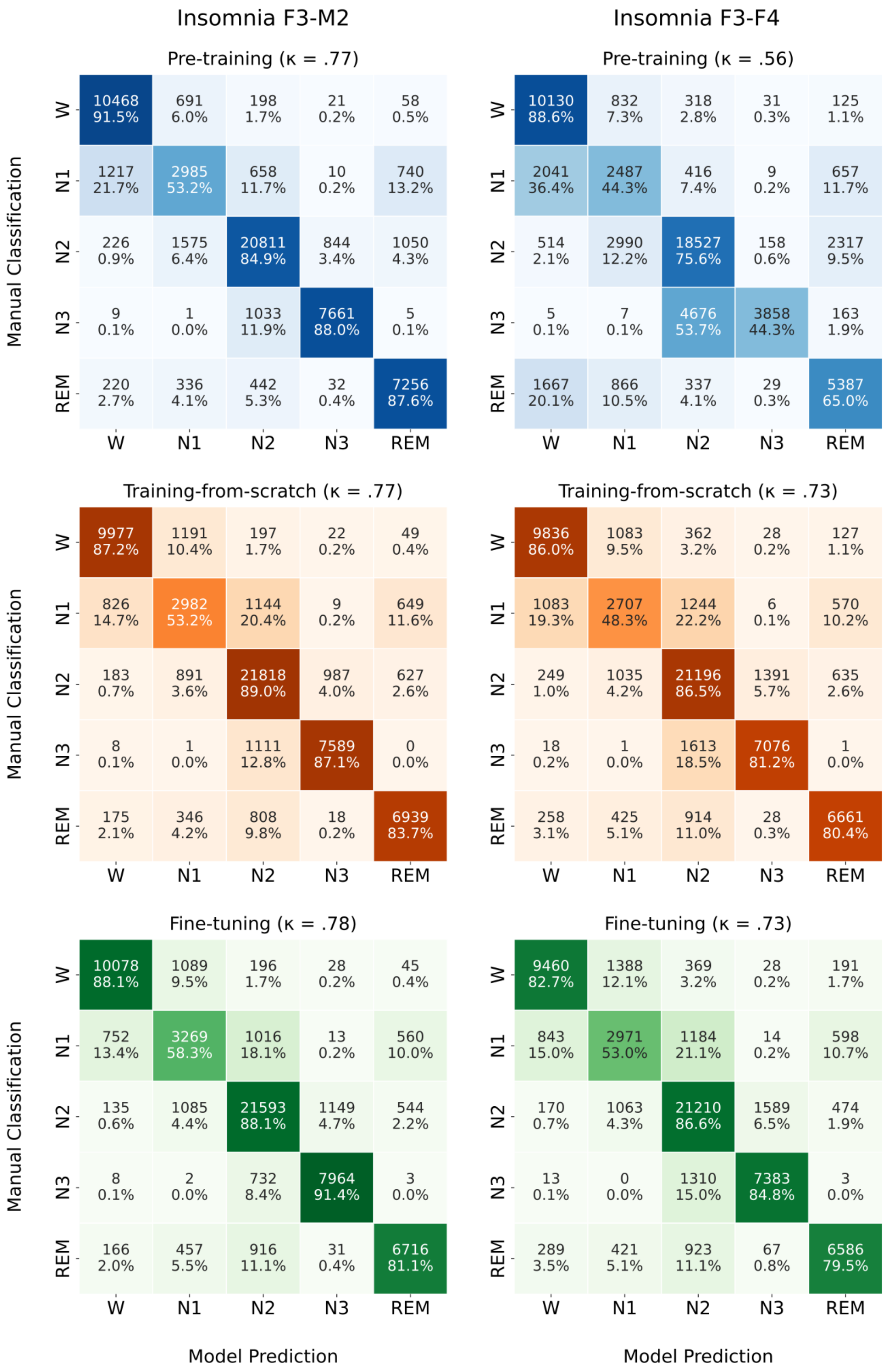

Supplementary Figure 3. Confusion matrices between manual and model classification for the training strategies in the Insomnia F3-M2 (left) and F3-F4 (right) datasets. Absolute number of epochs and percentage accuracy are given. Darker hue indicates higher accuracy.

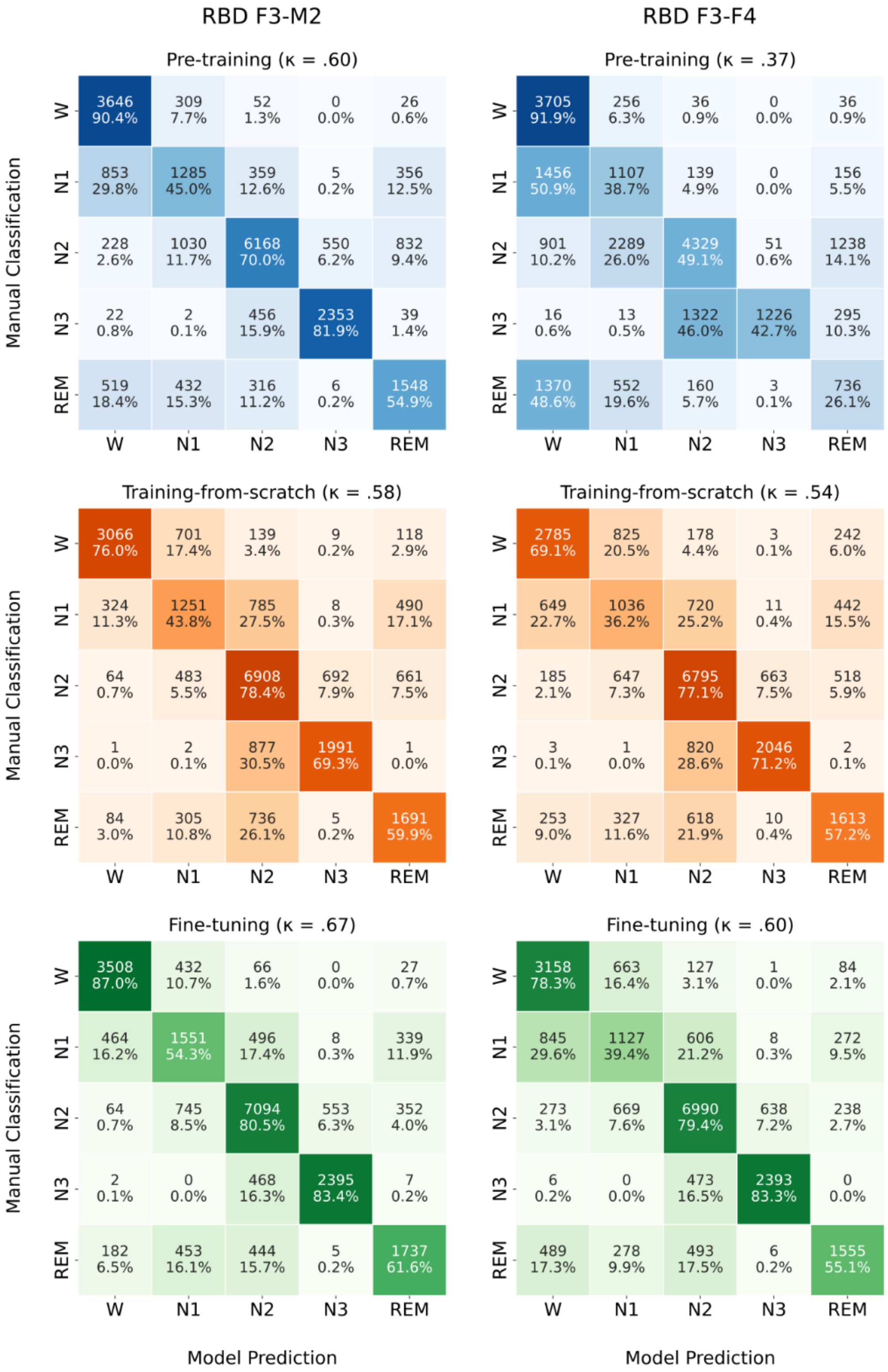

Supplementary Figure 4. Confusion matrices between manual and model classification for the training strategies in the RBD F3-M2 (left) and F3-F4 (right) datasets. Absolute number of epochs and percentage accuracy are given. Darker hue indicates higher accuracy.
